# Quantitative assessment of disease severity and rating of barley cultivars based on hyperspectral imaging in a non-invasive, automated phenotyping platform

**DOI:** 10.1186/s13007-018-0313-8

**Published:** 2018-06-08

**Authors:** Stefan Thomas, Jan Behmann, Angelina Steier, Thorsten Kraska, Onno Muller, Uwe Rascher, Anne-Katrin Mahlein

**Affiliations:** 1 INRES-Plant Protection and Plant Diseases, University Bonn, Bonn, Germany; 2 IBG2: Plant Sciences, Forschungszentrum Jülich GMBH, Jülich, Germany; 3 Field Lab Campus Klein-Altendorf, University Bonn, Bonn, Germany; 4 Institute of Sugar Beet Research (IfZ), Göttingen, Germany

**Keywords:** Hyperspectral imaging, Phenotyping platform, Greenhouse, High-throughput, Disease rating, Simplex Volume Maximization, Support Vector Machine

## Abstract

**Background:**

Phenotyping is a bottleneck for the development of new plant cultivars. This study introduces a new hyperspectral phenotyping system, which combines the high throughput of canopy scale measurements with the advantages of high spatial resolution and a controlled measurement environment. Furthermore, the measured barley canopies were grown in large containers (called Mini-Plots), which allow plants to develop field-like phenotypes in greenhouse experiments, without being hindered by pot size.

**Results:**

Six barley cultivars have been investigated via hyperspectral imaging up to 30 days after inoculation with powdery mildew. With a high spatial resolution and stable measurement conditions, it was possible to automatically quantify powdery mildew symptoms through a combination of Simplex Volume Maximization and Support Vector Machines. Detection was feasible as soon as the first symptoms were visible for the human eye during manual rating. An accurate assessment of the disease severity for all cultivars at each measurement day over the course of the experiment was realized. Furthermore, powdery mildew resistance based necrosis of one cultivar was detected as well.

**Conclusion:**

The hyperspectral phenotyping system combines the advantages of field based canopy level measurement systems (high throughput, automatization, low manual workload) with those of laboratory based leaf level measurement systems (high spatial resolution, controlled environment, stable conditions for time series measurements). This allows an accurate and objective disease severity assessment without the need for trained experts, who perform visual rating, as well as detection of disease symptoms in early stages. Therefore, it is a promising tool for plant resistance breeding.

## Background

Phenotyping is a necessary and time intensive step in the process of breeding disease resistant crops [1–3]. Visual rating by humans, the common non-destructive method of crop phenotyping, has the disadvantage of being a time-consuming, subjective process, which requires experts or trained personnel. The application of optical sensors is a promising approach to overcome the drawbacks of manual visual rating, as—with an adequate analysis algorithm—it is objective and can be automated, while allowing non-invasive measurements directly in greenhouses and fields [4–6]. Hyperspectral imaging (HSI) combines these advantages with ability to derive information about a large number of plant traits. Hyperspectral sensors have already been shown to be successfully integrated into automated measurement systems in greenhouses and fields [7, 8].

Hyperspectral sensors capture the reflectance characteristics of object in large number of wavelength bands. Similar to RGB cameras, they measure the light which is reflected at the measurement target, but they are sensitive in a larger area of the electromagnetic spectrum [9]. As a result, hyperspectral imaging cameras measure so called hyperspectral datacubes, which show the spatial dimensions of the acquired image and additionally a spectral dimension with the reflectance values per wavelength [9]. Hyperspectral imaging has been applied in multiple studies for biotic and abiotic stress detection in plants [10–13], as well as pathogen resistance assessment [14, 15].

However, experiments which are focused on disease detection at the earliest stages in pathogenesis are mostly performed as basic research in the laboratory on leaf scale. In contrast, field studies tend to focus on the detection of diseases at later stages of pathogenesis. In less controlled environments, environmental factors prove to be challenging for accurate hyperspectral measurements. As a result, detecting small symptoms at early stages of pathogen infection is more challenging. Changing light conditions during the measurements are the major environmental factor, reducing the data quality. Other factors, such as wind and rain, play a minor role [16–18]. As hyperspectral cameras with the highest spatial and spectral resolutions available to date tend to be push/whisk broom scanners, the process of image acquisition takes a certain amount of time [9]. During this process, the measurement accuracy is dependent on stable environmental conditions. Furthermore, the angle between incoming light, plant and sensor has influence on the measurement results [19, 20].

These problems multiply, when plant canopies are measured instead of leaves. In a dense canopy, the different leaves have individual angles to the light source and hyperspectral sensor. Furthermore, leaves are on different layers in the canopy. This leads to varying distances between measured leaves, sensor and illumination. Main effects are that leaves are less illuminated due to shadowing of the upper canopy layers and multiple scattering at surrounding leaves occurs [16, 21].

Common high throughput field hyperspectral measurement experiments are barely influenced by these factors, as they are either performed airborne or with non-imaging sensors, averaging the effects of canopy diversity over multiple leaves/plants [22, 23]. Although those procedures have shown to be successful in field monitoring and assessment of disease spread, they lack the spatial resolution to accurately rate the disease severity in early phases of infection and pathogenesis on plants.

Currently available hyperspectral measurement systems focus either on high measurement throughput like canopy measurements on the field with little regard to changes in the environmental factors [8, 24], or on plant/leaf level measurements under highly controlled environmental conditions with low throughput [13, 25, 26]. Both approaches are well suited for their fields of application.

However, the hyperspectral measurement system, which is introduced in this study, offers a new scale, specifically for phenotyping applications in resistance breeding. As it will be shown in this article, the different light conditions in plant canopies prove to be challenging for modern data analysis approaches even under nearly ideal measurement conditions. The proposed measurement system combines the high throughput of canopy based measurements in fields with the controlled measurement environment of laboratory setups in order to achieve stable data acquisition over the whole time course of disease development.

A greenhouse based phenotyping system, which is based on hyperspectral imaging, has been developed. The system works by growing plants in larger containers (Mini-Plots), which create a field like situation. Each Mini-Plot provides enough space in area and soil depth to grow a canopy consisting of 360 barley plants in similar density as they would be grown in the field. A soil depth of 61 cm allows a more natural development of the plants root systems when compared to commonly used pots. The combination of these factors allows for phenotyping experiments in greenhouses under conditions that resemble those of actual field experiments. The location of the measurement system inside a greenhouse has the innate advantage, that the environmental conditions during the experiment can be controlled at any time. Thereby, the system combines the advantages of phenotyping test plots in the field with the possibility of reliable measurements on a daily basis. This circumvents the problem of plants, which have been grown in pots in the greenhouse, showing different phenotypes compared to being grown under field conditions, due to stable environmental conditions and limited root development [27].

Halogen lamps, equipped with diffusors, are implemented in the measurement system, providing stable and diffuse light conditions for the hyperspectral camera. A transportable curtain is attached to exclude natural light, that may interfere with the measurement process. The system can perform automated measurements, allowing for a relatively high measurement throughput with minimal human effort. These factors summarize to the system being a valuable middle ground between field measurements under natural conditions, and low throughput measurements in highly controlled environments. The combination of tightly controlled environment and high measurement throughput with hyperspectral imaging shows the high potential of the presented system for phenotyping applications in resistance breeding.

To the author’s knowledge only two comparable systems exist at the time of this publication. Joalland et al. [28] designed a microplot based system, where the response of sugar beet plants to *Heterodera schachtii* inoculation was evaluated with different measurement methods. The microplots, containing three sugar beet plants per plot, were covered with a mobile dark box with a halogen lamp to provide equal light conditions before the average spectrum of the plants was collected with a non-imaging spectrometer. Busemeyer et al. [29] introduced the field based measurement system BreedVision, which can be moved over small plots and perform measurements with multiple sensors. A cover for the whole system provides shading and avoids direct solar radiation influencing the measurements. The system includes a hyperspectral imaging system to measure plant moisture and nitrogen content, which works with a spatial resolution of 3 × 5 mm. Compared to these systems, the Mini-Plot based system presented in this study features a reduced canopy effect through diffuse light conditions and a higher spatial resolution, which is important for the early detection of disease symptoms.

The system was tested by evaluating six barley cultivars with different disease susceptibility to powdery mildew. Over the course of the experiment, it could be shown that the gathered hyperspectral data allows an early detection of powdery mildew infection, as well as an accurate estimation of the disease severity for each barley cultivar per measurement day. The estimated values from the hyperspectral data analysis was consistent with the results of visual rating for each cultivar. Furthermore, it was possible to show the spatial distribution and spread of the pathogen over the barley plots during the time of the measurements.

## Methods

### Mini-Plot phenotyping greenhouse

**Fig. 1:**
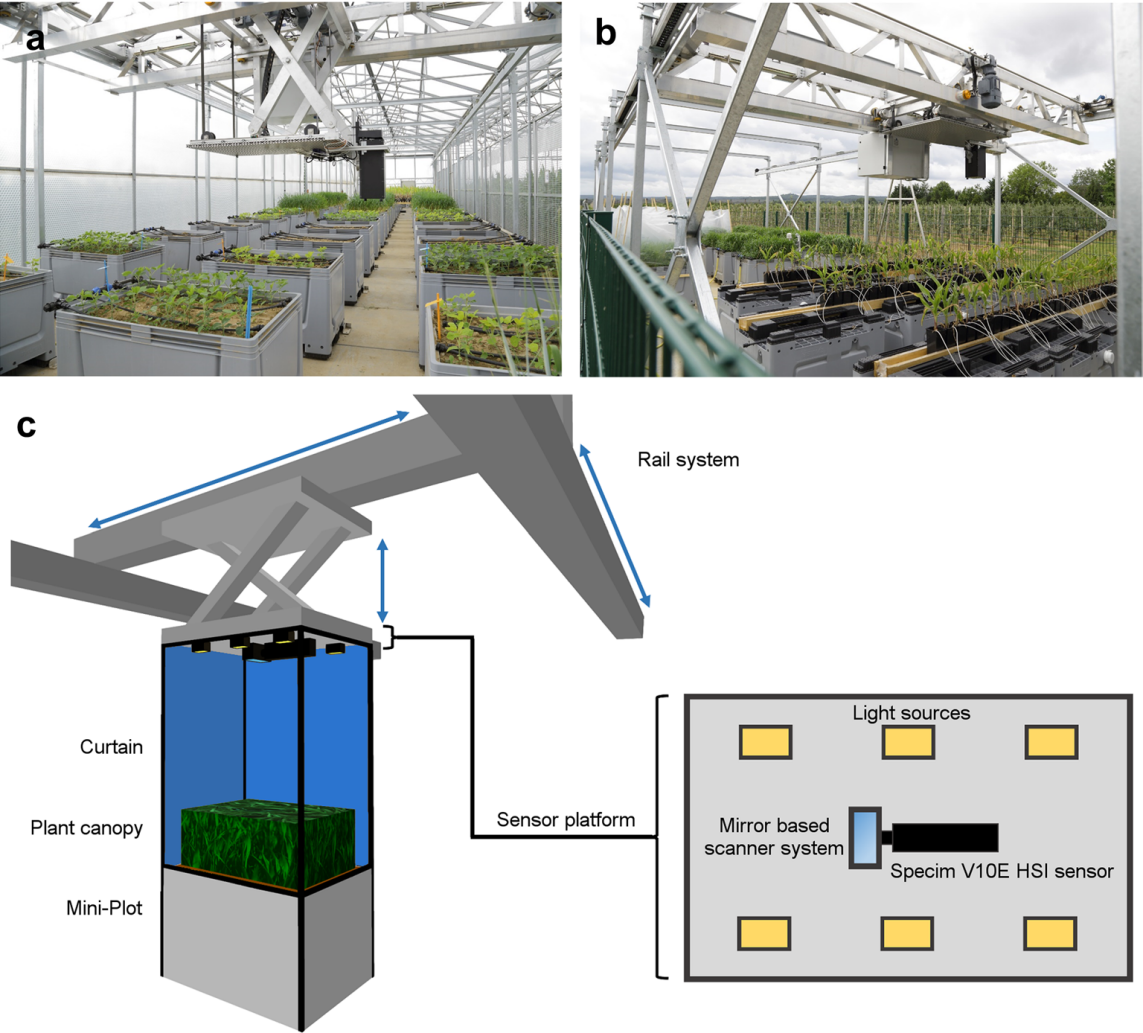
Phenotyping greenhouse in Campus Klein-Altendorf with Mini-Plot facility, interior (**a**) and exterior (**b**) compartments. Schematic representation of the hyperspectral phenotyping system (**c**). The rail system of the Mini-Plot facility in combination with diffuse artificial light sources and a curtain allows for automatic measurement approaches under highly controlled environmental conditions. The combination of Specim V10E hyperspectral imaging (HSI) sensor and mirror based scanner system enables fast, high-resolution measurements of the entire Mini-Plot

The hyperspectral measurements in this study have been performed in the ‘Mini-Plot’ facility at Campus Klein-Altendorf of Bonn University, which was developed by the Forschungszentrum Jülich. The facility consists of a large enclosed greenhouse compartment and a fenced-in outside area, where 120 large planting containers, so called Mini-Plots, can be placed. 90 Mini-Plots can be placed inside the greenhouse, while another 30 can be placed in the outside area (Fig. 1a, b). The experiments of this study were performed solely on Mini-Plots inside the greenhouse to be weather independent during the measurement series. An automated sensor positioning system facilitates the precise and robotized positioning of a sensor platform (Fig. 1). This allows for the cultivation of relevant crop species in small canopies, while above-ground plant traits can automatically be measured by a modular sensor positioning system, which can be equipped with a portfolio of phenotyping sensors (Fig. 1).

Each Mini-Plot is a commercial 535 L plastic container (inside size 111 × 71 × 61 cm; AUER Packaging, Belgium), that can be filled with local soil or other desired substrates, according to the goal of the experiments. An automated drip irrigation system is attached to each Mini-Plot allowing individual computer-controlled watering. Drainage is enabled by a loose gravel filling at the bottom and a valve in each container, the excess water can be quantified on request. Additionally, multiple environmental sensors are placed in the Mini-Plot area to monitor the environmental conditions and potential gradients; monitoring includes irradiance, air temperature and humidity, as well as soil moisture and temperature. A weather station (Vaisala) is located in the fenced-in area outside the greenhouse to monitor weather conditions and to avoid outside measurements during bad weather conditions.

The automatic positioning system was developed by the Forschungszentrum Jülich in cooperation with Otte Metallbau GmbH & Co Kg (Harkebrügge, Germany) in partnership with Atlantique Automatisierungstechnik GmbH (Ihlow, Germany). A stable and motorized x, y, z rail based traversing unit is installed in the greenhouse and the outside area. This traversing unit moves a universal base plate, on which various sensors of up to 50 kg can be attached. The base-plate is moved 2 meters above the containers (2.8 m above ground) and can be positioned with an accuracy of 2 cm using fixed positioning elements at the x and y axis. The universal baseplate (including sensors) can be lowered in z direction to facilitate close range measurements; technically the base plate can be lowered by 1 m, i.e. bringing sensors in proximity to the plant canopy.

The system is controlled by an in-house developed application based on LabVIEW (National Instruments, USA). Measurements can be scheduled throughout the day, triggering different sensors. The base functions of the system (movement of the traversing unit, switching of the watering valves) are controlled by the Programmable Logic Controller (PLC). These functions can be controlled by the user directly as well. In the automated mode, the communication between the PLC (Siemens, Germany) of the system and the sequence control application (LabVIEW) is facilitated using an OPC server (LabVIEW). The sequence application is commanding the measurement routine, which has been programmed by the user. Within a schedule file, the time when a sensor is triggered, the position/plot and the distance to the container can be configured. It is possible to measure each plot with separate sensors during a single measurement sequence. The acquired data is stored on the acquisition computer located at sensor platform and transferred daily to the server.

### Plant materials and pathogens

Six barley cultivars with different susceptibility to *Blumeria graminis* f. sp. *hordei* (*Bgh*), based on assessment of the official German cultivar list (Descriptive Variety List; Bundessortenamt, Hanover, Germany), were used in the experiments. The used cultivars are (respective disease susceptibility rating in bracelets): Tocada (7; KWS Lochow GmbH, Bergen, Norway), Grace (7; Ackermann Saatzucht GmbH & Co. KG, Irlbach, Germany), Milford (4; Saatzucht Josef Breun GmbH & Co. KG, Herzogenaurach, Germany), Gesine (4; NORDSAAT Saatzuchtgesellschaft, Halberstadt OT Langenstein, Germany), Eileen (2; KWS Lochow GmbH) and Irina (2; KWS Lochow GmbH). Each cultivar was sown into two Mini-Plots, with 360 seeds per Mini-Plot to simulate barley growth under field conditions. The distribution of the cultivars in the phenotyping greenhouse was randomized to avoid location affects. Directly after sowing, Plantosan fertilizer (20% N, 10% P_2_O_5_, 15% K_2_O, 6% MgO, 2% S, Wilhelm Haug GmbH &Co. KG, Germany) was applied according to the manufacturer’s description to each Mini-Plot. The experiment was performed from 16. 09. 2016–02. 12. 2016, with low air temperatures in the greenhouse, following the procedure established in the preliminary experiment from 09. 11. 2015–18 .01. 2016. The barley cultivars were cultivated for 4 weeks until sufficient surface cover to perform the experiments was reached. Mini-Plots, which showed development of powdery mildew symptoms prior to inoculation, were treated with sulfur (fungicide Kumulus containing 800 g/kg sulfur, BASF, Germany) to prevent further symptom development. Symptomatic leaves were removed from the respective Mini-Plots and sulfur was washed off multiple times before inoculation. Two days before inoculation with *Bgh*, half of the Mini-Plots were treated with the fungicide Vegas (containing 53.1 g/l cyflufenamid, BASF, Germany), to serve as negative control for the experiment. The other half of the Mini-Plots were inoculated with conidia of *Bgh* field isolate from Bonn by shaking heavily infested plants above the Mini-Plots.

### Manual rating of disease development per barley cultivar

Both control and inoculated Mini-Plots of each barley cultivar were visually assessed on every measurement day. Plant and plant disease development were assessed and documented with RGB images. RGB images were taken from above the Mini-Plot—to achieve the same viewing angle as the hyperspectral imaging sensor. Furthermore, close up RGB images of areas with disease symptoms or other anomalies—like necrotic lesions at resistant cultivars—were acquired.

At the last measurement day (30 dai) a visual rating of the inoculated Mini-Plots from each barley cultivar was performed [30]. Three classes of disease severity were defined: Low (up to 5% of the plot showing powdery mildew symptoms), moderate (5% to 20% of the plot showing powdery mildew symptoms) and severe (over 20% of the plot showing powdery mildew symptoms) disease severity.

### Hyperspectral imaging measurement on canopy scale

The hyperspectral reflectance measurements were performed with a Specim V10E hyperspectral push broom sensor (Spectral Imaging Ltd., Oulu, Finland), which was mounted on the rail system based sensor platform in the phenotyping greenhouse (Fig. 1). The Specim V10E sensor provides hyperspectral image acquisition in the visual (400–700 nm) and near infrared (700–1000 nm) region of the electromagnetic spectrum with a spectral resolution of approximately 2.8 nm. During the measurements, a spatial resolution of 0.3 mm was obtained in a measurement distance of 80 cm. A mirror scanner (Spectral Imaging Ltd., Oulu, Finland) was used to change the field of view of the push broom sensor in order to acquire two dimensional images.

Additionally, 6 halogen lamps (POWLI010 Halogen Floodlight 150 Watt; Varo, Belgium) were symmetrically distributed on the sensor platform to achieve homogenous lighting conditions (Fig. 1c). The glass cover of each halogen lamp was replaced by a frosted, highly heat resistant glass cover to diffuse light. Ambient natural light was excluded by the use of a light-proof white curtain covering both the sensor platform and the measured Mini-Plot (Fig. 1c). The curtain also provides additional scattering of the light from the halogen lamps, leading to a more homogenous illumination of the measurement samples.

All Mini-Plots were measured in a time-series experiment from 1 dai (days after inoculation) to 30 dai. For each measurement, a barium sulfate 99% reflectance white reference bar (Spectral Imaging Ltd., Oulu, Finland) was measured before the measurement of plant canopy, providing known illumination intensity values for image normalization. After each measurement (both white reference and plant canopy), a dark current measurement of the internal camera noise was performed with the same exposure time as the previous image, respectively, eliminating inaccuracies during image normalization.

An additional measurement with identical observation parameters as described above was performed 2 h before the inoculation of the plants with powdery mildew, in order to ensure that the fungicide treatment of the plants had no effects on the spectral signatures. The confirmatory results were in accordance with previous experiments [13]. Due to the lack of a pathogen, the data has not been included in the analyzed time series.

### Analysis of the hyperspectral dataset

ENVI 5.1 + IDL 8.3 (ITT Visual Information Solutions) was used to normalize the hyperspectral images against the known values of the white reference standard, while subtracting the dark currents of both images. The normalized images were further smoothed by the application of the Savitzky-Golay filter [31] to the spectral domain, in order to reduce noise in the spectral profiles of the images. Areas of the images which were not covered by plants, as well as areas with extremely low light intensity in the lower canopy were masked during the preprocessing of the images. Furthermore, all images were cropped to the designated measurement area of the experiment.

**Fig. 2:**
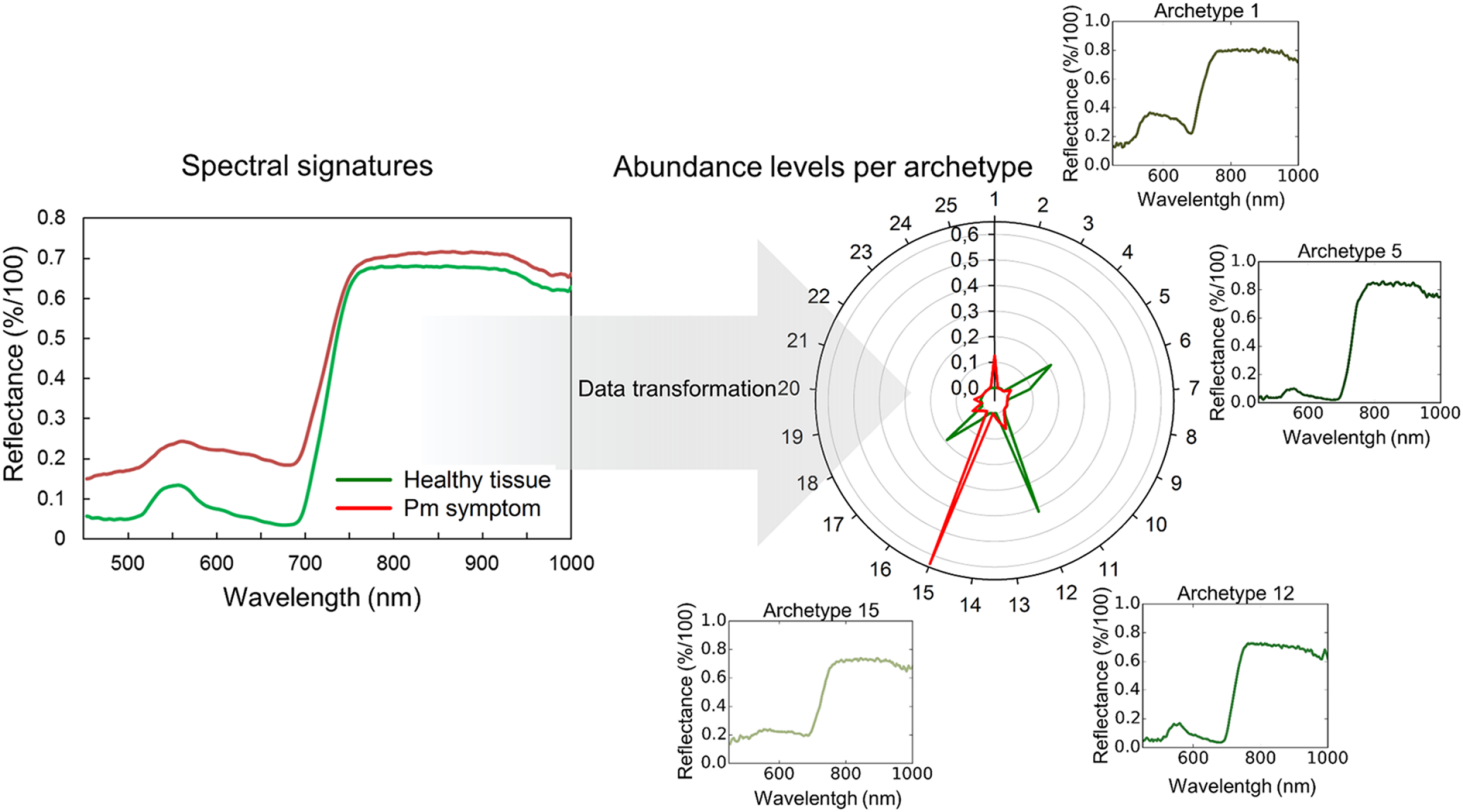
Spectral signatures and abundance maps of healthy plant tissue and powdery mildew symptoms (mean values of 50 pixels, each). The spectral signatures (left) represent the mean reflectance of the pixels over the spectral measurement area of the sensor. The abundance map (right) shows the representation of the same mean values based on the abundance of the pixels with the 25 archetypes, which were selected during the transformation of the dataset with the Simplex Volume Maximization. Archetypes with high correlation to healthy or symptomatic tissue are shown separately, each archetype is a real spectral signature from the original hyperspectral dataset (colors of archetype spectral signatures represent the color of the corresponding pixels, which would be visible to the human eye). *Pm* powdery mildew

Simplex Volume Maximization (SiVM) was then applied on all preprocessed images. SiVM is an unsupervised data analysis method, which selects extreme hyperspectral signatures of the dataset as archetypes for a re-parameterization of the whole dataset (Fig. 2) [32]. The application of SiVM leads to a reduction in size of the dataset and a pre-classification of the data based on the abundance level of each generated archetype [33]. In this study, the SiVM algorithm was performed with 25 archetypes for the entire dataset of hyperspectral images, including control and inoculated images of each barley cultivar. Thereby, the size of the dataset was reduced to ~ 27% of the original size. The matrix factorization toolbox PYMF 0.3 [32, 34] was used for this approach.

The SiVM transformed dataset was then classified into healthy tissue, disease symptoms and background by a non-linear Support Vector Machine algorithm (SVM) [35]. The applied SVM uses radial basis function as kernel function to determine non-linear discriminant functions. As a supervised method, it is based on training data, i.e. manually selected samples as examples for each class, which were selected for each class by an expert at an unmistakable development state. The required hyperparameters were determined using a cross-validation based grid optimization. LIBSVM 3.21 was used [36]. Training data for the SVM classification was selected and annotated—based on a combination of pseudo RGB representation and spectral information of the hyperspectral dataset—by an expert. The inoculated Mini-Plot of barley cultivar Tocada at 22 dai was chosen to collect training data, due to its representative powdery mildew symptoms in different development stages and canopy layers. After manual selection of pixels with disease symptoms and healthy tissue in the different canopy layers, the gathered data was used as reference for the above described SVM classification. In order to access the accuracy of the resulting SVM classification the image of the inoculated Mini-Plot of barley cultivar Grace at 22 dai was manually annotated as described above. The manual annotation was compared with the automatic annotation of the SVM classification result for that image, showing the accuracy of the automated data analysis over different cultivars despite the limited training data.

## Results and discussion

### Visual observations and manual assessment of the spectral dataset

Both control and inoculated plants developed slower than usual over the course of the experiment and did not produce ears. This was expected due to low temperatures in the greenhouse compared to the usual growth temperatures for summer barley. The slower development of the barley plants and the powdery mildew symptoms enabled long term measurements of the disease progression. Thereby, it was possible to confirm the performance of the non-invasive measurement setup over a prolonged period of time. No plant damage except the effects of the powdery mildew infestation could be detected during the measurement period of 30 days.

The plants in the control Mini-Plots showed no signs of powdery mildew infection from 1 to 24 dai. Starting at 26 dai, the control plants of the susceptible cultivars Milford, Grace and Tocada showed first signs of powdery mildew symptoms at the edges of the Mini-Plot. Meanwhile, the inoculated plants of the cultivars Milford, Tocada and Grace showed sporadic symptoms from 12 dai on the edges of the Mini-Plot and first symptoms of strong powdery mildew infection in the measurement area at 14 dai (Table 1). At 18 dai, moderate infection in early stages could be observed at inoculated Mini-Plots of cultivars Milford, Tocada and Grace, which did increase up to 30 dai. The cultivars Tocada and Grace showed moderate powdery mildew symptoms (Table 1). However, cultivar Milford showed the highest disease severity (severe), despite being listed with moderate susceptibility in the official German cultivar list. Cultivar Eileen showed few visible symptoms at 14 dai, while having a low susceptibility for powdery mildew infection (Table 1). Cultivar Eileen, unlike the three aforementioned cultivars, showed no significant development of powdery mildew symptoms over the course of the experiment. Despite being listed as moderately susceptible to powdery mildew, cultivar Gesine showed no symptoms until 22 dai (Table 1). From this point on, the disease severity steadily increased until the end of the measurements at 30 dai. Some necrotic lesions became visible on the plants, starting at 14 dai. The cultivar Irina showed no signs of powdery mildew infection over the course of the experiment. Severe necrotic lesions over the leaves were visible, starting at 14 dai (Table 1). Overall, the different cultivars showed powdery mildew development and disease severity according to their general rating. Only the cultivars Gesine and Milford interacted different than expected from their assessed susceptibility. Gesine showed a surprisingly high resistance against powdery mildew, with a notable delay in symptom development compared to other susceptible cultivars. Meanwhile, Milford, despite being listed moderately susceptible, showed the strongest disease severity and symptom development of all cultivars. These results were coherent for the Mini-Plot experiment, as well as preliminary experiments in the greenhouse the Mini-Plot facility and microscopic analysis. The explanation is most likely the specific interaction of the cultivars with the used *Bgh* isolate.

**Table 1:** Manual rating of disease progression per barley cultivar over the course of the experiment (2016; – = no symptoms appeared)

Barley cultivar	Disease susceptibility*	First symptoms (dai)	Relative disease severity at 30 dai	Necrotic lesions visible (dai)
Tocada	7	14	Moderate	–
Grace	7	14	Moderate	–
Milford	4	14	Severe	–
Gesine	4	22	Low	14
Eileen	2	14	Low	–
Irina	2	–	–	14

*According to rating of the official German cultivar list

**Fig. 3:**
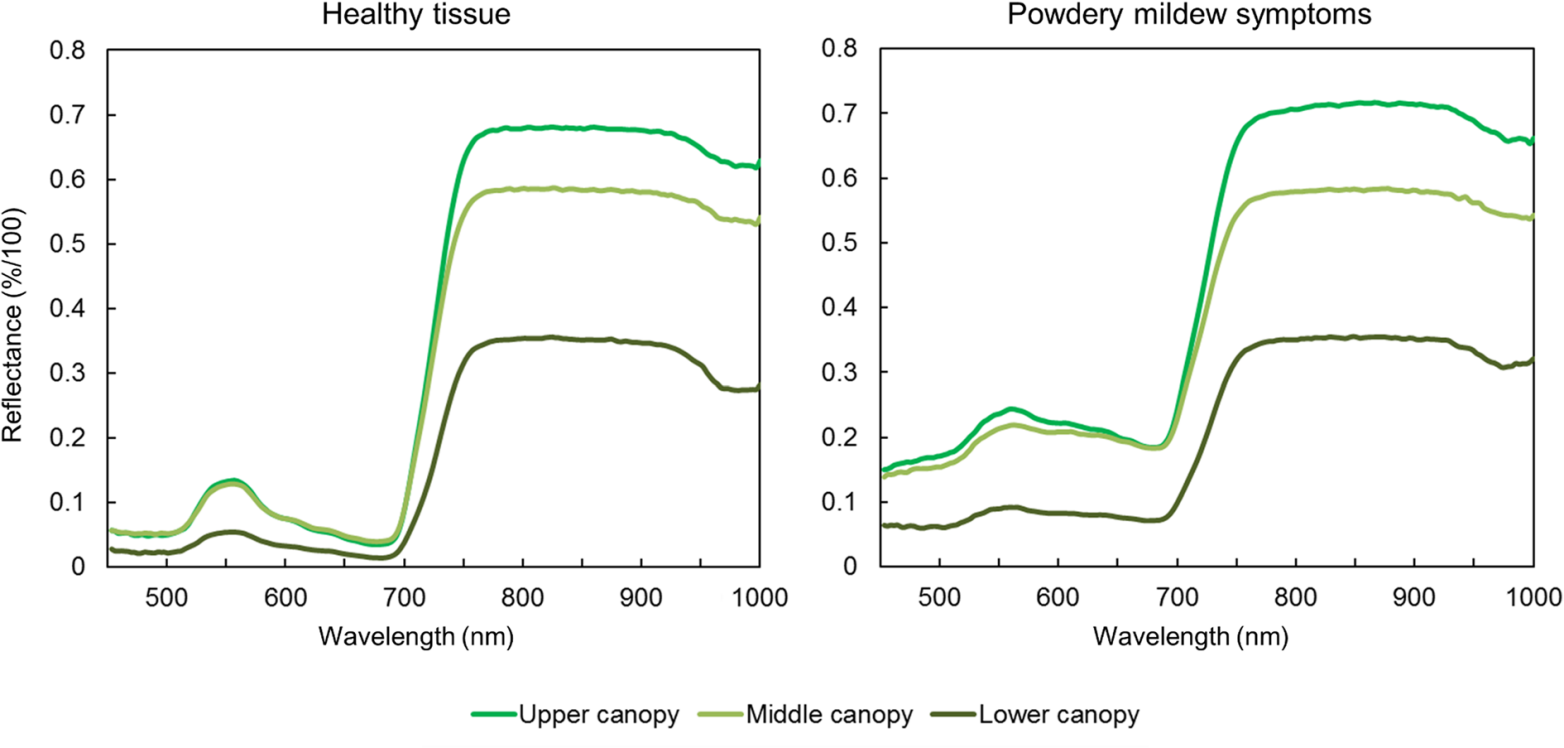
Spectral signatures of mean values from 30 pixels on canopy levels, differing in distance to the sensor and illumination system, for both healthy and symptomatic tissue

Average spectra of pixels with powdery mildew symptoms and healthy tissue were extracted, unveiling the characteristic changes in the plants spectral signature upon powdery mildew infection when comparing pixels in corresponding canopy layers. Pixels in different canopy layers showed differences in the intensity of their spectral signatures over all measured wavelengths, as changes in the intensity of the incoming light and shadows have a strong effect on the data. The spectral signature of infected leaves shows mostly a general increase in intensity, with a pronounced increase of reflection at 650–700 nm (Fig. 3). Both pixels with powdery mildew symptoms and healthy tissue have the highest variety for different canopy layers in the near infrared area between 750 and 1000 nm (Fig. 3). The observed variance in the near infrared area of the spectral profiles can be explained by the high sensitivity of near infrared reflection measurements to distance and angle of the target. Thomas et al. [13] showed, that even slight changes of leaf angles in framed leaves lead to high variances in the near infrared part of the spectrum. In the case of canopy measurements, the differences in leaf angle and distance to the camera are greatly increased. The distance and angle of the white reference—serving as standard value for 100% light reflectance—is fixed during the measurement. This leads to an increase in the calculated reflectance in the near infrared area of the spectrum if leaves are closer to the sensor than the white reference. These effects were not corrected in this study, as the study focuses on relative detection of powdery mildew symptoms, rather than their spectral characterization. The changes in the spectral profile of the pixels with disease symptoms are typical for powdery mildew infestation, due to the symptoms are visible as a layer of white mycelia on the leaf with minimal influence on the leaf structure [13]. Thereby, it proved to be difficult to distinguish spectral signatures of symptomatic and healthy areas, which are located in different layers of the canopy and thereby show differences in the intensity of the reflected light (Fig. 3).

Hyperspectral images acquired under artificial light conditions showed to be superior to images acquired under natural light conditions. The diffuse light of constant intensity did reduce the effects of the canopy structure on the hyperspectral signatures considerably and increased the overall image quality. The image quality, with diffuse light conditions and a spatial resolution of 0.3 mm, was sufficient to distinguish symptoms at 12 dai, when they were first visible with the human eye. These results show the importance of controlled environmental conditions. It was possible to perform stable measurement series over prolonged time periods with comparable results. Additionally, distinct advantages of equally distributed and diffuse lights for an improved measurement quality on the canopy scale could be observed. The results of this study strongly suggest a notable increase in measurement accuracy under diffuse light conditions through a reduced impact of the canopy architecture on the light intensity differences of the individual canopy layers. Furthermore, the measurement system of this study offers a high spatial resolution (0.3 × 0.3 mm pixel size) when compared with similar phenotyping systems in the field (Busemeyer et al. [29] with 3 × 5 mm pixel size) and more comparable to leaf scale laboratory setups [13, 16, 25].

### Analysis of the acquired hyperspectral data through SVM

Due to the high data variability across the different layers of the canopy and the extensive amount of the gathered hyperspectral dataset, it was necessary to perform advanced data analysis methods. The application of SiVM significantly reduced the size of the data (from 234 to 62 GB) and pre-classified the dataset based on existing, extreme spectral signatures. This approach did increase both speed and accuracy of the SVM based classification. Due to the nature of hyperspectral imaging, techniques for data size reduction are an important part of a functional phenotyping system. When taking into consideration that each image can easily reach the size of several gigabytes, large scale phenotyping experiments are not only requiring a lot of storage space, but also require modern data analysis methods, which tend to be time consuming [20]. SiVM has the advantage that the structure of the data is not lost, as each spectrum is classified based on existing data instead of abstract variables [37]. This allows for a simplified representation with increased separability of the acquired data and a considerable reduction in processing time using the SiVM processed dataset in further data analysis methods.

**Fig. 4:**
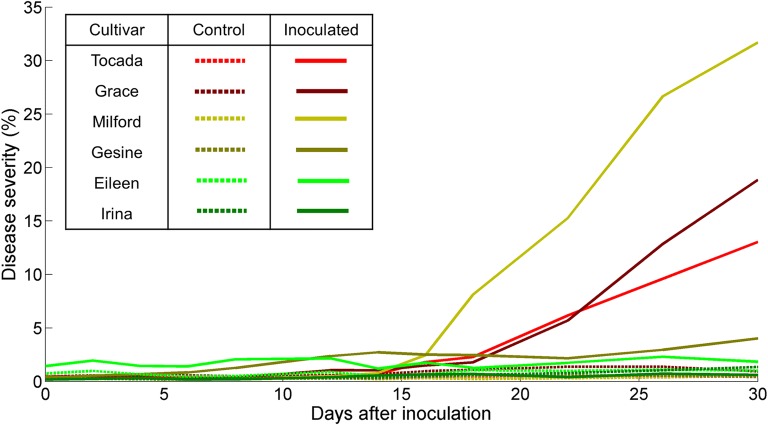
Disease severity of the different barley cultivars over the course of the experiment. The disease severity was estimated based on percentage of pixel, which were classified as containing powdery mildew symptoms after Simplex Volume Maximization (SiVM) and following supervised classification by Support Vector Machines (SVM)

It was possible to separate pixels showing healthy tissue, powdery mildew symptoms and background into different classes through the combined classification with SiVM and SVM. Classification of the control Mini-Plots showed less than 2% of the pixels in the images being classified as diseased for all cultivars whereas up to 31% of the pixels are predicted as infected for the inoculated plots (Fig. 4). Meanwhile, the automatic disease severity estimation of the infected plants of all cultivars in the Mini-Plots, based on the SVM results, show matching results to the visual observations of the disease progression per cultivar at 30 dai (Fig. 4). Furthermore, the progression of the disease severity can be accurately tracked across every measurement date during the experiment. Validation of the SVM classification data was based on its application and comparison with a manually labelled hold-out dataset. The dataset was derived from images of the inoculated Mini-Plot of cultivar Grace at 22 dai to verify cross cultivar accuracy. The results show an accuracy of 94.83% for the automatic SVM classification. The results of this study show that the proposed hyperspectral phenotyping system is able to accurately assess the disease severity of each cultivar. Additionally, it is possible to monitor the exact progression of the disease symptoms for each plot at any time during the time series measurement. Combined with the high throughput of the canopy level measurements, the system allows for a quick and objective estimation of barley cultivar susceptibility to powdery mildew. In this study, a high potential of the system to be used for fully automated measurements in the future has been confirmed.

**Fig. 5:**
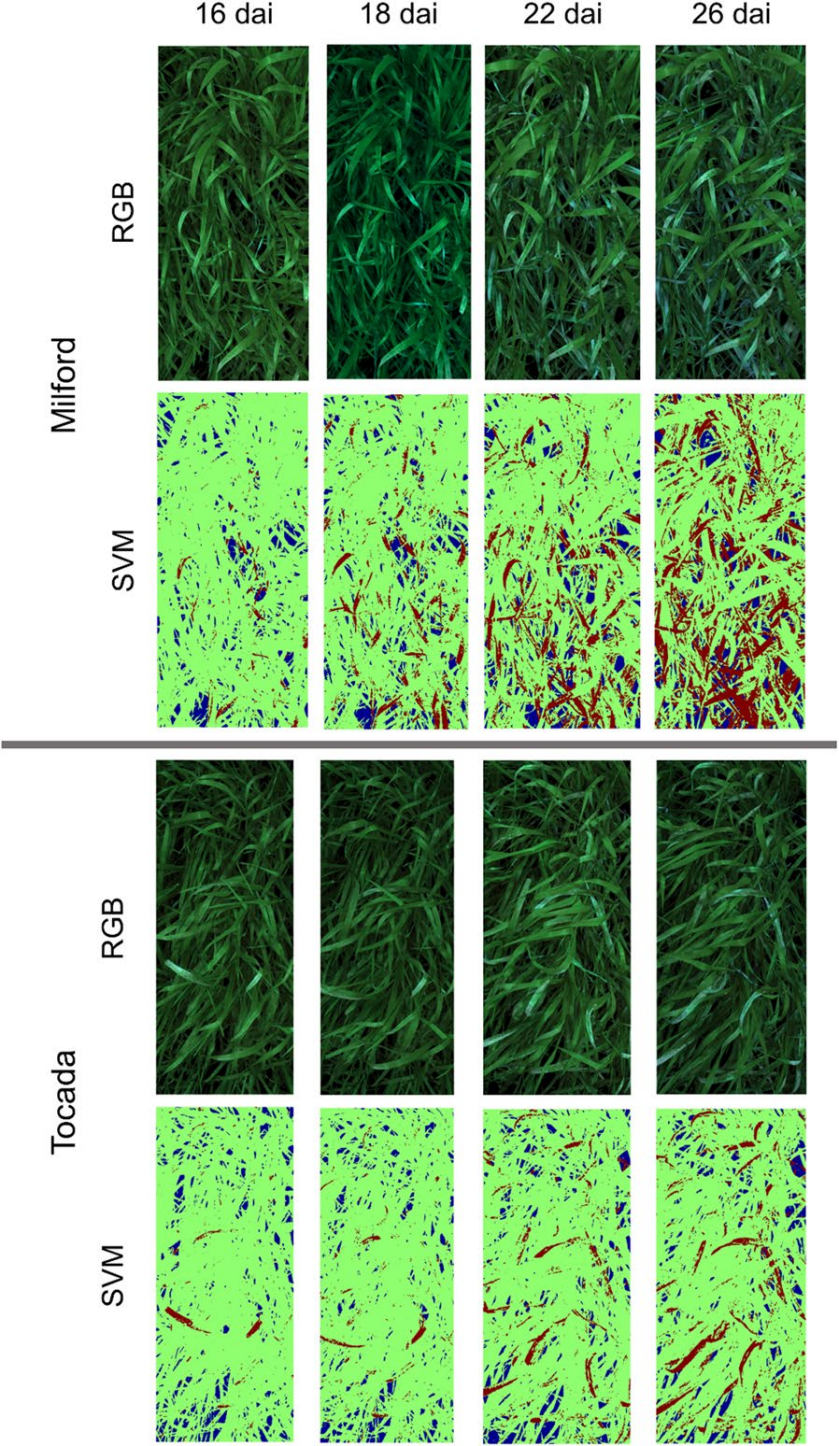
Spatial distribution of powdery mildew infestation development over the course of the experiment for highly susceptible cultivars Milford and Tocada through pseudo RGB images and false color images of Support Vector Machines (SVM) classification (*green* healthy tissue, *red* powdery mildew symptoms, *blue* background)

The results of the SVM classification can also be visualized on the pixel scale for each hyperspectral image of the cultivars over the course of the experiment, providing spatial information about disease outbreak and spread over the course of the experiment (Fig. 5). In Fig. 5, the disease progression of the two cultivars with the highest disease severity (Milford and Tocada) is shown. The differences in disease patterns for the cultivars from the first visual symptoms at 14 dai up to the final stages of powdery mildew infestation can be readily assessed and analyzed for phenotyping purposes. Furthermore, the SVM classification is able to detect first disease symptoms at 12 dai, when they first appeared at the Tocada and Milford cultivars. Due to the similarities of powdery mildew symptoms and healthy tissue with specular reflections, it is difficult to detect such early symptoms without misclassifications. However, such first symptoms contribute only to a small degree to the proportional disease severity estimation. A specific and quantitative evaluation of the early detection was beyond the scope of the experiment and was not specifically regarded in the analysis. Unlike the results of Thomas et al. [13], which detected powdery mildew on barley at leaf scale under laboratory conditions, it was not possible to detect infestation before visible symptoms appeared in the current study. This can be explained as a manual annotation of training data was required due to the SVM classifier. As it was impossible to acquire training data from pixels with powdery mildew infestation before they became visible to the human eye, the SVM algorithm could not be trained to search for these effects. Kuska et al. [14] were able to select powdery mildew infected areas before visible symptoms appeared. However, this was possible due to tracing position on the leaf from later stages in the experiment, when symptoms have become visible. Due to the leaf movement in the partially opened greenhouse, this technique could not be transferred to the current study. Nevertheless, the results show, that powdery mildew symptoms at early stages can be detected in the canopy with the current setup, providing valuable information for resistance breeding.

### Automated assessment of necrosis due to resistance against powdery mildew of cultivar Irina

**Fig. 6:**
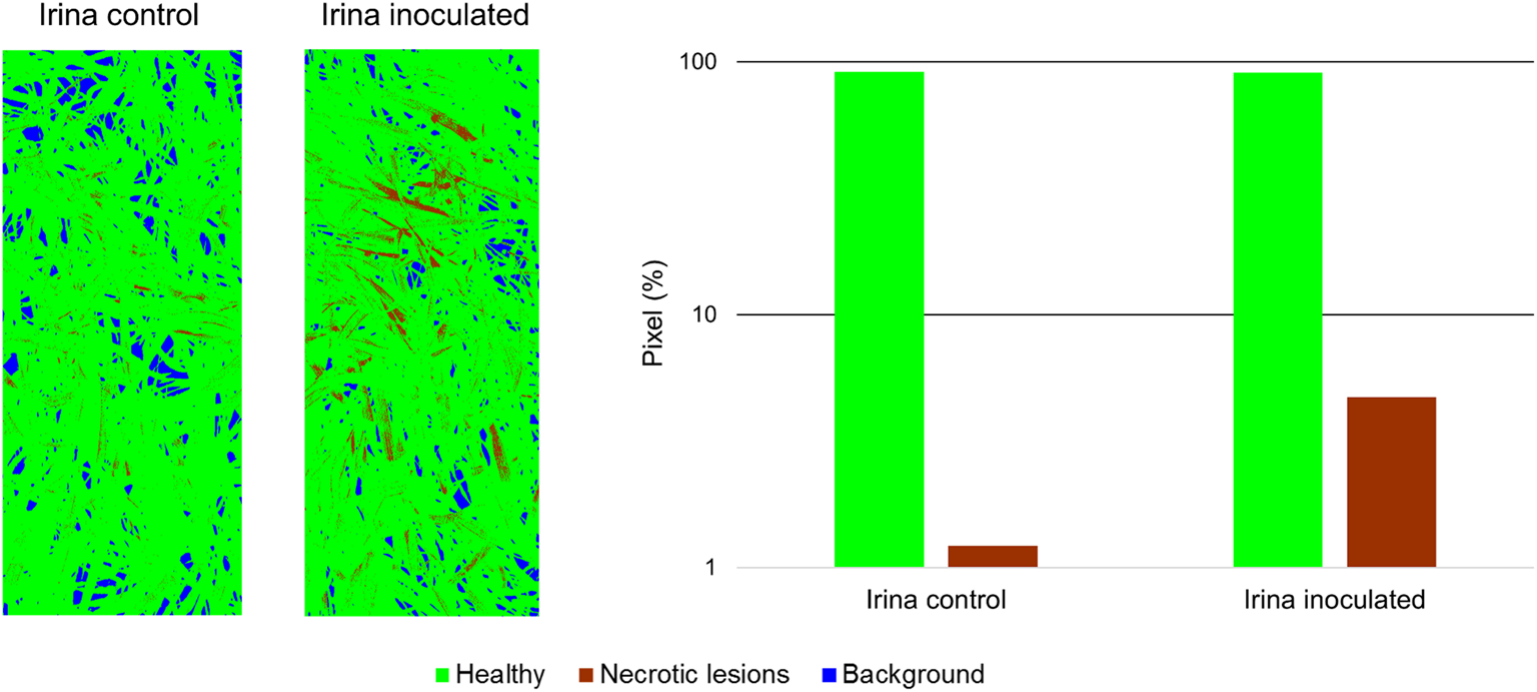
Classification of healthy tissue and tissue expressing necrotic lesions as response to powdery mildew infection at the resistant barley cultivar Irina 2 days after inoculation. False color pictures show the spatial distribution of necrotic lesions over the Mini-Plots of both control and inoculated plants. Additionally, the percentage of total pixels in the image being classified as showing tissue with HR for both plots is shown as bar diagram. The inoculated plot shows a significantly increased number of pixels, which are classified as expressing necrotic lesions

Necrotic spots on the leaves of barley cultivar Irina could be spotted in the canopy at 14 dai. Microscopic analysis could identify papillae formation which prevents cell wall penetration of powdery mildew haustoria, as well as hypersensitive response (HR) as response to successful penetration of epidermis cells. Due to the intensity of necrotic lesions on the inoculated plants, it was possible to create training data, based on the plants in the inoculated Mini-Plot of cultivar Irina at 14 dai and perform SVM classification for healthy tissue and tissue with necrotic lesions. As shown in Fig. 6, it was possible to differentiate inoculated and control plants of cultivar Irina, based on the increase of necrotic lesions in response to powdery mildew inoculation. The training of the SVM classification had to be performed with a small number of samples, which could be identified by the authors due to large amounts of necrotic cells creating visible discolorations on the plant leaves. Nevertheless, these observations are promising for the application of the proposed hyperspectral phenotyping system for the direct detection of resistance reactions in plants as response to pathogen attack. For such experiments, it is possible to reduce the distance between camera and plants inside the Mini-Plots. This would lead to the required higher spatial resolution at the cost of a reduced measurement throughput and more extreme observation geometries. By the development of modern, more compact hyperspectral cameras, the measurement setup can be further simplified in the future [38].

## Conclusion

The proposed hyperspectral phenotyping system was designed to enable the accurate measurements of basic research leaf level hyperspectral experiments, usually performed under controlled conditions in the laboratory, at a high throughput with environmental conditions as close to the field as possible. The conducted study shows that, despite the intrinsic difficulties of performing measurements on canopy scale, the system is able to detect disease symptoms at early stages and allow detailed assessment of disease severity and progression over extended periods of time. The achieved results are objectively derived and the measurement process is non-invasive, allowing for repeated measurements without interference with the plants development. Moreover, it was also possible to detect powdery mildew resistance induced necrosis for one of the resistant cultivars, which is a valuable addition for applications in resistance breeding programs. The use of modern data analysis methods enabled automatic extraction of the results and it was possible to analyze the entire dataset, containing over 234 GB of information, by creating a set of training data from a single hyperspectral image. The proposed hyperspectral measurement system improves efficiency and accuracy of phenotyping procedures and would be a valuable addition in plant resistance breeding programs.

## Abbreviations

HSI: hyperspectral imaging
PLC: programmable Logic Controller
*Bgh*: *Blumeria graminis* f. sp. *hordei*
dai: days after inoculation
SiVM: Simplex Volume Maximization
SVM: Support Vector Machines
HR: hypersensitive reaction
Pm: powdery mildew
